# A novel diabetes typology: towards precision diabetology from pathogenesis to treatment

**DOI:** 10.1007/s00125-021-05625-x

**Published:** 2022-01-04

**Authors:** Christian Herder, Michael Roden

**Affiliations:** 1 Institute for Clinical Diabetology, German Diabetes Center (Deutsches Diabetes-Zentrum/DDZ), Leibniz Center for Diabetes Research at Heinrich-Heine-University Düsseldorf, Düsseldorf, Germany; 2 Department of Endocrinology and Diabetology, Medical Faculty and University Hospital Düsseldorf, Heinrich-Heine-University Düsseldorf, Düsseldorf, Germany; 3 German Center for Diabetes Research (DZD), Partner Düsseldorf, München-Neuherberg, Germany

**Keywords:** Clustering, Complications, Diabetes subgroups, Personalised medicine, Precision medicine, Reclassification, Review

## Abstract

The current classification of diabetes, based on hyperglycaemia, islet-directed antibodies and some insufficiently defined clinical features, does not reflect differences in aetiological mechanisms and in the clinical course of people with diabetes. This review discusses evidence from recent studies addressing the complexity of diabetes by proposing novel subgroups (subtypes) of diabetes. The most widely replicated and validated approach identified, in addition to severe autoimmune diabetes, four subgroups designated severe insulin-deficient diabetes, severe insulin-resistant diabetes, mild obesity-related diabetes and mild age-related diabetes subgroups. These subgroups display distinct patterns of clinical features, disease progression and onset of comorbidities and complications, with severe insulin-resistant diabetes showing the highest risk for cardiovascular, kidney and fatty liver diseases. While it has been suggested that people in these subgroups would benefit from stratified treatments, RCTs are required to assess the clinical utility of any reclassification effort. Several methodological and practical issues also need further study: the statistical approach used to define subgroups and derive recommendations for diabetes care; the stability of subgroups over time; the optimal dataset (e.g. phenotypic vs genotypic) for reclassification; the transethnic generalisability of findings; and the applicability in clinical routine care. Despite these open questions, the concept of a new classification of diabetes has already allowed researchers to gain more insight into the colourful picture of diabetes and has stimulated progress in this field so that precision diabetology may become reality in the future.

**Graphical abstract:**

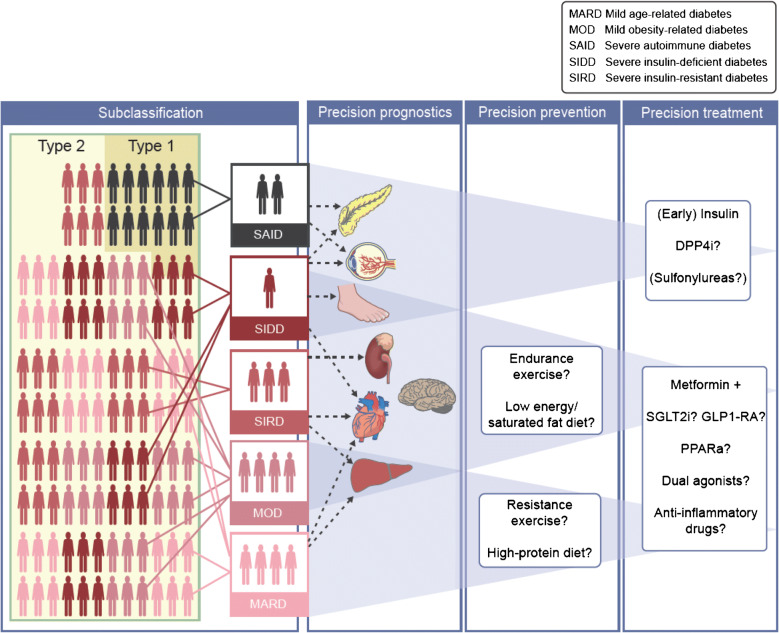

**Electronic supplementary material:**

The online version of this article (10.1007/s00125-021-05625-x) contains peer-reviewed but unedited supplementary material including a slide of the figure for download, which is available to authorised users.

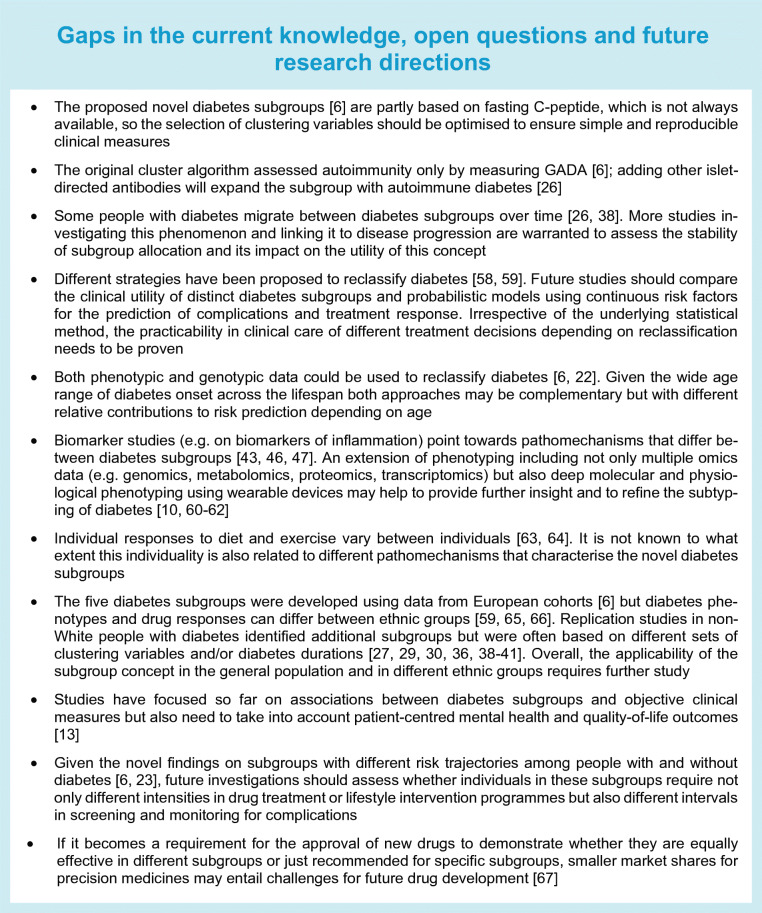



## Rationale for diabetes reclassification

The observation that people with diabetes have different phenotypes has led to repeated attempts to classify the main diabetes types [1, 2]. Electronic supplementary material (ESM) Table 1 summarises the key efforts in this evolution. People with diabetes show a broad variation in the main features of diabetes (i.e. insulin resistance and beta cell dysfunction [3–6]) as a result of the combined effects of (epi)genetic, environmental and lifestyle factors and their different contributions in different individuals. A recently proposed ‘palette model’ conceptualises the interaction of these factors [3]. People at risk of diabetes may have impairments in multiple processes such as islet development, islet function, autoimmunity, inflammation, insulin sensitivity, incretin activity and adipose tissue function (considered as ‘base colours’). Every individual is positioned somewhere within the spectrum of the phenotypic variation of each trait as determined by their genetic variation and non-genetic exposures modifying these processes, and the sum (or mixture) of all trait variations represents the overall estimate of metabolic health and diabetes status [3]. However, this pathophysiological heterogeneity is not captured by current position statements and guidelines for diagnosis and treatment of diabetes [7, 8].

Differences in risk factors and pathophysiological mechanisms are thought to drive the heterogeneity in preclinical abnormalities, prevalence of comorbidities and clinical complications already seen at diagnosis of diabetes [5, 9]. People with diabetes further vary in the progression of their disease and in the incidence of diabetes-related complications despite comparable glycaemic control.

Any reclassification effort should be seen as an example of precision medicine or ‘precision diabetology’ aiming to deconstruct the heterogeneity of diabetes. Advances in the management of monogenic forms of diabetes (neonatal diabetes, MODY) represent a successful proof-of-concept for a reclassification of diabetes [10]. Currently, however, relatively few people with diabetes are affected by monogenic diabetes so this may serve as an example for personalised medicine based on mutations in single genes [11] rather than the precision medicine approach that is required for type 1 and type 2 diabetes, which are both polygenic and multifactorial [12].

In the context of type 1 and type 2 diabetes, the ultimate purpose of precision diabetology is the development of stratified prevention and treatment for subgroups of people with different risk profiles. These options range from refined screening and monitoring intervals, recommendations for tailored lifestyle interventions, to targeted but not individualised drug treatment. Clinical benefits envisaged include fewer adverse effects and ideally a delay of the onset of diabetes and its complications, lower morbidity and mortality and an economic use of resources [13].

The aim of this review is to provide an up-to-date, concise overview of studies on diabetes reclassification, their implications, and also inherent practical and methodological challenges, with a specific focus on recent definitions of subgroups of type 2 diabetes and the risk of complications in these subgroups. With respect to subgroups and endotypes of type 1 diabetes, we would like to refer the reader to recent excellent reviews covering aspects of precision diabetology for this diabetes type [8, 14].

## Variability of disease presentation and progression

One approach to study the heterogeneity of diabetes relies on cohorts of people included at or shortly after the diagnosis of diabetes [5]. Even though the duration of hyperglycaemia before diabetes diagnosis is unknown, these cohorts allow the investigation of clinical characteristics that are not yet confounded by long-term excessive hyperglycaemia and pharmacological treatment. Examples are the German Diabetes Study (GDS [5]), the Verona Newly Diagnosed Type 2 Diabetes Study (VNDS [15]) and the All New Diabetics in Scania (ANDIS [6]) cohorts.

The comprehensive phenotyping in the GDS, using gold-standard methodology, demonstrated large interindividual differences in people within 12 months of their diagnosis of diabetes regarding insulin sensitivity, beta cell function, islet-directed autoantibodies, blood lipids and BP [5, 16]. The variability in disease severity or progression is reflected by differences between subgroups of type 2 diabetes regarding diabetes-related complications such as chronic kidney disease (CKD), distal sensorimotor polyneuropathy (DSPN), cardiovascular autonomic neuropathy (CAN), retinopathy and non-alcoholic fatty liver disease (NAFLD) [5, 16, 17].

The VNDS enrols people with type 2 diabetes within 6 months of their diagnosis. Assessment of diabetes-related complications indicated a high variability in their presentation and progression [15, 18]; the prevalence of one or more diabetes-related complications (CVD, nephropathy, DSPN, CAN, retinopathy) already present at study enrolment was found to be 49.2% [9].

The ANDIS cohort includes incident cases of diabetes and reported a marked prevalence of NAFLD at baseline (although only based on surrogate measurement by alanine aminotransferase) and the development of CKD, retinopathy and CVD in the first years after the diagnosis of diabetes [6].

In addition, people with diabetes differ with respect to inherited factors. The application of a polygenic risk score (PRS) based on >136,000 variants in the UK Biobank demonstrated a prevalence of type 2 diabetes of 1.2% and 11.2% in the lowest and highest 2.5% of the PRS distribution, respectively, revealing an almost tenfold difference [19].

Taken together, these studies highlight the substantial variability in the pathogenic and clinical characteristics of the large population of people commonly designated as having type 2 diabetes.

## Novel subtypes of diabetes reflecting differences in disease development and progression

### Reclassification methods

From a methodological perspective, different clustering algorithms have been used to reclassify people with diabetes [20–25]. Topology-based analysis [21] and Bayesian non-negative matrix factorisation clustering [22] are widely applied procedures for discovering groups of related observations (e.g. subgroups of people with diabetes) using high-dimensional data such as electronic medical records or omics data. Cluster analysis based on the *k*-means or the partitioning around medoids methods [6, 23] break large datasets up into subgroups by minimising the distance between data points labelled to be in a cluster and a point designated as the centre of that cluster. These methods require that the optimal number of clusters (*k*) needs to be known a priori (i.e. evaluated with other methods). In contrast, latent-class trajectory analysis is a longitudinal analysis method using repeated measures of dependent variables as a function of time to identify subgroups of people who differ in trajectories (e.g. in glucose response curves) [24, 25].

In addition to these methods, reclassification studies made use of datasets that differed widely in the type and number of variables. One clustering approach, using high-dimensional electronic medical records and extensive genotype data, identified three subtypes of type 2 diabetes enriched in CVD, nephropathy, retinopathy, neurological diseases and cancer [21]. A second approach used data for 94 type 2 diabetes-associated gene variants and 47 diabetes-related traits to subgroup genetic loci according to mechanistic pathways and to relate the clinical characteristics of people with type 2 diabetes to their genetic risk scores [22]. This study found two clusters of genetic loci related to insulin deficiency and three related to insulin resistance. Individuals with high genetic risk scores in the respective clusters also differed in obesity, lipids, hypertension, kidney function and CVD [22].

From a clinical perspective, clustering algorithms based on available patient data would be highly attractive. One study used latent-class trajectory analysis based on mixed-meal tolerance tests in people with newly diagnosed type 2 diabetes [25] and identified three subgroups based on their glucose response patterns. Thus, this method represents another classification approach closely related to insulin resistance and insulin secretion as the pathophysiological hallmarks of type 2 diabetes.

### Diabetes subgroups

The most frequently replicated study in this field used both hierarchical and *k*-means clustering in Swedish people with newly diagnosed diabetes, with the following six variables as input: GAD antibodies; age at diagnosis; BMI at diagnosis; HbA_1c_; and HOMA-2 estimates of insulin resistance and beta cell function calculated from fasting glucose and C-peptide [6]. The resulting subgroups (subtypes) were designated as severe autoimmune diabetes (SAID), severe insulin-deficient diabetes (SIDD), severe insulin-resistant diabetes (SIRD), mild obesity-related diabetes (MOD) and mild age-related diabetes (MARD) [6] (Table 1). This concept has been replicated in cohorts from Europe, North America and Asia despite varying disease duration since diabetes diagnosis [26–32]. The SAID subgroup comprises people who are otherwise classified as having type 1 diabetes (including those previously termed latent autoimmune diabetes of adults), whereas SIDD, SIRD, MOD and MARD represent novel entities of type 2 diabetes. The subgroups also differ with respect to prevalence and/or risk of complications (Table 1). In line with the predominant insulin deficiency, ketoacidosis at diagnosis is most frequent in SAID and SIDD [6]. Retinopathy, DSPN and CAN are observed most often in SIDD [6, 26], while CKD and NAFLD are most prevalent in SIRD [6, 26, 29]. Adjusted risk ratios for prevalent erectile dysfunction are highest for SIDD and SIRD [33]. Although the subgroups differ in their cardiovascular risk, these differences did not remain statistically significant after adjustment for age and sex in the ANDIS cohort [6] or after more comprehensive adjustment for multiple covariables in a Japanese cohort [29].

**Table 1:** Metabolic characteristics and diabetes-related complications of individuals in the novel diabetes subgroups

Diabetes subgroup	Metabolic characteristics	Diabetes-related complications
SAID	Early-onset diabetes Low BMI High HbA_1c_ Insulin deficiency Presence of GADA	Ketoacidosis at diagnosis [6] High risk of retinopathy [29] High incidence of CKD but dependent on baseline eGFR [28]
SIDD	Early-onset diabetes Low BMI High HbA_1c_ Insulin deficiency GADA negative	Ketoacidosis at diagnosis [6] High risk of retinopathy [6] Highest prevalence of DSPN [26] Highest prevalence of CAN [26] High prevalence of erectile dysfunction [33]
SIRD	Late-onset diabetes High BMI Most insulin-resistant GADA negative	Highest liver fat content, fatty liver index, NAFLD fibrosis score and prevalence of NAFLD [6, 26, 29] Highest risk for macroalbuminuria, CKD and end-stage renal disease [6, 26, 29] High risk of coronary event and stroke (dependent on age and sex) [6] High prevalence of erectile dysfunction [33]
MOD	Early-onset diabetes High BMI Intermediate insulin resistance GADA negative	Intermediate prevalence and risk of diabetes-related complications [6, 26]
MARD	Late-onset diabetes Low BMI GADA negative	High risk of coronary events and stroke (dependent on age and sex) [6]

Metabolic characteristics are based on European cohorts with newly diagnosed diabetes using GAD antibodies, age at diagnosis, BMI at diagnosis, HbA_1c_ and HOMA-2 estimates of insulin resistance and beta cell function calculated from fasting glucose and fasting C-peptide concentrations as clustering variables [6, 26]

GADA, GAD antibodies

### Subgroup variables

The clinical relevance of the novel subgroups has been assessed in multiple cohorts including ethnically diverse populations that lack some of the aforementioned clustering variables (most often C-peptide measurements). Partial replication of the subgroup classification and differential risk of complications was reported in cohorts from Europe [34, 35], the USA [36, 37], Mexico [38], Latin America and the Caribbean [39], India [40] and China [41] and in large international trial populations (DEVOTE/LEADER/SUSTAIN-6 [42]) (Table 2). At present it is not known whether C-peptide or insulin are required to identify SIRD, so it would be informative to compare different combinations of clustering variables (e.g. with and without C-peptide or insulin) in the same cohorts, to better understand their relevance for reclassification.

**Table 2:** Overview of clustering studies using alternative demographic and clinical variables to identify subgroups of diabetes

Cohort characteristic	Clustering variables	Subgroups	Specific findings	Ref.
VNDS, Italy (739 with T2D)	Age, BMI, HOMA-2 estimates of beta cell function and insulin resistance	SIDD MARD OIRD EOD	Replication of SIDD and MARD OIRD comprising MOD and SIRD MARD associated with CVD Highest HbA_1c_ after 14-month follow-up in SIDD	[34]
Three cohort studies from Europe: Hoorn DCS; GoDARTS; ANDIS (15,940 people with T2D, within 2 years of diagnosis)	Age, BMI, HbA_1c_, random or fasting C-peptide, HDL-cholesterol	Five distinct T2D subgroups	Three subgroups could be mapped back to the original ANDIS clusters (SIDD, SIRD, MOD) Two subgroups (MD and MDH related to MARD) Progression to insulin fastest for SIDD and slowest for MDH	[35]
MASALA and MESA multi-ethnic cohorts from USA (1293 people with diabetes; mean diabetes duration 5.7 years)	Age at diagnosis, BMI HbA_1c_, HOMA estimates of beta cell function and insulin resistance	Five T2D subgroups: older age, severe hyperglycaemia, severe obesity, younger age at onset; requiring insulin medication use	Older age most common subgroup for all race/ethnicities apart from South Asians Severe hyperglycaemia subgroup most frequent in South Asians Risk for renal complications and subclinical CVD differed by subgroup and by race/ethnicity	[36]
Look AHEAD (5145 overweight/obese people with T2D and 10 years of lifestyle intervention or control group)	Age at diagnosis, BMI, WC, HbA_1c_	Four subgroups: by older age at diabetes onset; poor glucose control; severe obesity; younger age at diabetes onset	Interaction between lifestyle intervention and diabetes subgroups for three composite cardiovascular outcomes Increased cardiovascular risk for people in subgroup with poor glucose control randomised to lifestyle intervention	[37]
NHANES (USA) and four Mexican cohorts (1758 people with T2D in NHANES; 9887 people with T2D in the open-population Mexican cohorts)	Models based on different combinations of years since diagnosis, BMI, HbA_1c_, HOMA-2 estimates of beta cell function and insulin resistance, fasting plasma glucose, METS-IR, METS-VF, age at diabetes onset	Four subgroups: obesity-related; insulin-deficient; insulin-resistant; age-related	Risk of retinopathy highest for insulin-deficient subgroup and lowest for obesity-related subgroup Subgroup transitions observed after 3 months, 1 year and 2 years	[38]
Thirteen cohort studies from nine countries in Latin America and the Caribbean (8361 people with T2D)	Age, sex, BMI, WC, systolic/diastolic BP, T2D family history	Four clusters: Cluster 0, highest BP; Cluster 1, highest BMI and WC, highest proportion of positive family history of diabetes; Cluster 2, most beneficial risk profile; Cluster 3, highest age	Heterogeneous distribution of clusters across countries	[39]
Electronic medical records of a tertiary diabetes centre, India (19,804 people with T2D; diabetes duration <5 years)	Age at diagnosis, BMI, WC, HbA_1c_, triacylglycerols, HDL-cholesterol, C-peptide (fasting and stimulated)	Four clusters: Cluster 1, SIDD; Cluster 2, IROD; Cluster 3, CIRDD; Cluster 4, MARD	SIDD and MARD similar to diabetes subgroups in other populations IROD and CIRDD unique to Asian Indian population IROD showed highest BMI and highest C-peptide levels CIRRD showed lowest age of onset, highest serum triacylglycerols, highest risk for kidney disease	[40]
Retrospective clinic-based study sample, PR China (5414 people with T2D; mean diabetes duration 8.6 years)	Age at diagnosis, BMI, HbA_1c_, HOMA-2 estimates of beta cell function and insulin resistance, GADA; additional model with triacylglycerols and uric acid	Replication of SAID, SIRD and MARD when using the original six clustering variables Replication of SAID, SIDD, SIRD, MOD and MARD and identification of novel subgroups (UARD, IRD) when all clustering variables were used	Higher risk for retinopathy, peripheral neuropathy, hypertension and CKD for SIRD (vs IRD) Higher risk for retinopathy and diabetic foot for SIDD (vs IRD)	[41]
Three global cardiovascular outcomes trials: DEVOTE, LEADER, SUSTAIN-6 (20,274 people with T2D; follow-up of 2.0–3.8 years)	Age at diagnosis, BMI, HbA_1c_	Identification of four subgroups: clusters A–D	Differences between clusters for major adverse cardiovascular events, cardiovascular death, nephropathy and severe hypoglycaemia when comparing subgroups in at least one cohort	[42]

CIRDD, combined insulin-resistant and deficient diabetes; DCS, Diabetes Care System; DEVOTE, Trial Comparing Cardiovascular Safety of Insulin Degludec vs Insulin Glargine in Patients With Type 2 Diabetes at High Risk of Cardiovascular Events; EOD, early-onset diabetes; GADA, GAD antibodies; GoDARTS, Genetics of Diabetes Audit and Research; IRD, inheritance-related diabetes; IROD, insulin-resistant obese diabetes; LEADER, Liraglutide Effect and Action in Diabetes: Evaluation of Cardiovascular Outcome Results; MASALA, Mediators of Atherosclerosis in South Asians Living in America; MD, mild diabetes; MDH, mild diabetes with high cholesterol; MESA, Multi-Ethnic Study of Atherosclerosis; METS-IR, Metabolic score for insulin resistance; METS-VF, metabolic score for visceral fat; NHANES, National Health and Nutrition Examination Survey; OIRD, obese insulin-resistant diabetes; SUSTAIN-6, Trial to Evaluate Cardiovascular and Other Long-term Outcomes With Semaglutide in Subjects With Type 2 Diabetes; T2D, type 2 diabetes; UARD, uric acid-related diabetes; WC, waist circumference

Only a few studies have explored biomarkers and pathways underlying differences between subgroups that could determine susceptibility to diabetes-related complications. Given the role of lipid metabolism in diabetes, it is noteworthy that serum triacylglycerol levels were found to be highest and HDL-cholesterol levels lowest in SIRD, while there were no differences in total or LDL-cholesterol [26, 29, 43]. Circulating levels of angiopoietin-like protein 8 (ANGPTL8), a regulator of lipid metabolism, were higher in SIDD, SIRD and MARD than in MOD [44]. However, these differences were not adjusted for the clustering variables.

### Subgroup differences in inflammation

Circulating levels of high-sensitivity C-reactive protein (hsCRP) were highest in SIRD and MOD [29]. The association of circulating triacylglycerols and inflammatory processes with insulin resistance is in line with the uniform mechanism underlying common insulin resistance in humans [45]. A multimarker approach in the GDS found that 23 biomarkers of inflammation differed between the subgroups, with biomarker levels in general being highest in SIRD and lowest in SIDD [46]. After adjustment for the clustering variables, serum caspase-8 (CASP-8), S100 calcium-binding protein A12 (EN-RAGE) and IL-6 showed at least one pairwise difference between the subgroups. The association between inflammation and insulin resistance reflects the contribution of inflammation-related processes to SIRD, whereas inflammatory processes appear less relevant in SIDD [46]. A second study in this cohort showed that the SIRD subgroup also had high leucocyte numbers and the highest CD4^+^ T cell percentages, thereby demonstrating different immune cell frequencies between subgroups and highlighting the proinflammatory characteristics of SIRD [47]. Of note, studies on autoimmune diabetes identified both genetic and epigenetic determinants of T cell function, with effects on gene expression [48, 49]. (Epi)genetic variation and its impact on transcriptomes in immune cells will require more detailed analyses with respect to relevance in disease aetiology in the other subgroups.

### Genetic predisposition

There is evidence that the subgroups may differ in their associations with gene variants predisposing to diabetes. The HLA SNP rs2854275 showed the same association with SAID as with type 1 diabetes in previous studies, but not with SIDD, pointing towards different aetiologies of insulin deficiency in the subgroups [6]. Both the *TCF7L2* SNP rs7903146, which is known for its association with type 2 diabetes, and a genetic score for type 2 diabetes were associated with SIDD, MOD and MARD but not with SIRD [6]. A genetic risk score for insulin secretion was associated with MOD and MARD (and nominally with SIDD) but again not with SIRD [6]. This suggests a more pronounced role for genetic predisposition to SIDD, MOD and MARD and a stronger role for environmental determinants in SIRD. Of note, individuals with SIRD were more frequently carriers of the G allele of rs738409 in *PNPLA3*, the gene encoding patatin-like phospholipase domain-containing-3, which is characterised by its positive association with hepatic fat content [43] and may contribute to the relationship between SIRD and progression of NAFLD to fibrosis [6, 26, 29]. Thus, genetic analyses corroborate the difference between SAID and the other subgroups but suggest unique mechanisms that might distinguish SIRD from SIDD, MOD and MARD.

At present, the only study integrating genetic, metabolomic, lipidomic and proteomic data to compare diabetes subtypes was based on different clustering variables (age, BMI, HbA_1c_, HDL-cholesterol, and random or fasting C-peptide). SIRD showed the most distinct molecular signature, mostly related to insulin resistance, lipids and inflammation [50].

## Subgroups of individuals with different risk of progression to type 2 diabetes and complications

Trajectory analyses show that changes in metabolic and inflammation-related biomarkers start >10 years before the onset of type 2 diabetes [51–53]. Therefore, it is also of high clinical relevance to identify subgroups of individuals at different risk for diabetes and for complications, which may start even before the manifestation of diabetes. Latent-class trajectory analysis using OGTTs in people without diabetes revealed four subgroups that differed in anthropometric, metabolic and inflammation-related variables [24], but this study did not analyse diabetes-related complications.

A recent study in a cohort of individuals at elevated risk of type 2 diabetes explored the pathophysiological heterogeneity before clinical diabetes onset [23]. Participants from the Tübingen Family Study (TUEF) and Tübingen Lifestyle Intervention Program (TULIP) underwent clustering based on OGTT, MRI (body fat distribution, liver fat), serum lipids and a PRS for type 2 diabetes. This study found six subphenotypes differing in diabetes-related variables: 1, low risk; 2, very low risk; 3, beta cell failure; 4, low-risk obese; 5, high-risk insulin-resistant fatty liver; 6, high-risk visceral fat nephropathy [23]. Results were replicated in the Whitehall II cohort using a reduced set of clustering variables. Overall, clusters 3, 5 and 6 showed higher glucose levels at baseline but only clusters 3 and 5 had an increased incidence of type 2 diabetes. Clusters 3, 5 and 6 featured the highest CKD risk and higher intima–media thickness, and clusters 5 and 6 had the highest all-cause mortality. Data from Whitehall II indicated that individuals from the low-risk clusters 1, 2 and 4 transitioned to MOD and MARD with diabetes onset, whereas individuals from the high-risk cluster 6 transitioned to SIRD [23]. Thus, clustering approaches can also identify subphenotypes with respect to glycaemic, renal, cardiovascular and all-cause mortality risk, corresponding to previous findings for overt diabetes [6, 26].

## Translation into clinical practice: therapeutic implications

**Fig. 1:**
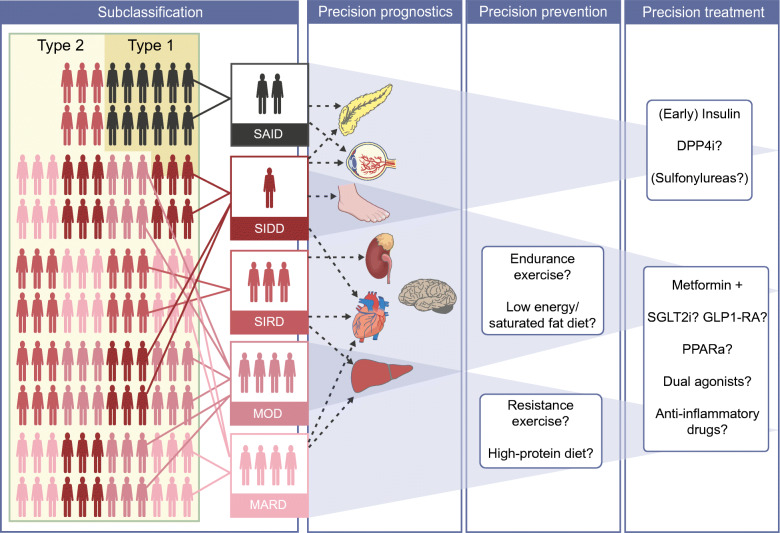
Possible future implications of precision diabetology based on the novel diabetes subgroups. Although the utility of the concept needs to be evaluated in RCTs, one may speculate on the potential implications of a new (sub)classification of diabetes for tailored diagnosis, prevention and treatment. Individuals in the different diabetes subgroups differ in their susceptibility to developing specific complications. The different (pathophysiological) phenotypes may also differ in their response to lifestyle-related and pharmacological strategies. SAID requires early introduction of insulin supplementation, whereas SIDD may also benefit from a dipeptidyl peptidase 4 inhibitor (DPP4i) or, when cost is a major issue, a sulfonylurea. SIRD and MOD would benefit from medication that induces weight loss (SGLT2i, GLP-1RA, dual agonist) or also addresses risk of CVD or nephropathy (SGLT2i, GLP-1RA). Providing that safety and efficacy have been established, new insulin sensitisers (e.g. peroxisome proliferator activator receptor agonists) or anti-inflammatory drugs could also improve targeted treatment of SIRD. On the other hand, individuals with MARD should receive treatments avoiding weight loss and sarcopenia (e.g. protein-balanced diets and moderate resistance training). PPARa, peroxisome proliferator activator receptor agonist. This figure is available as a downloadable slide

RCTs are required to evaluate the clinical relevance of reclassification efforts. Until data from subgroup-specific RCTs are available, it is only possible to investigate in cohort studies [6, 29] or intervention trials, such as A Diabetes Outcome Progressive Trial (ADOPT) [28], whether individuals allocated to subgroups differ in their treatment at study baseline or their treatment responses, respectively (Table 3). Importantly, the high frequency of individuals without initial glucose-lowering treatment, the low frequency of insulin use and the shortest time to reach the HbA_1c_ target were similar for SIRD, MOD and MARD and correspond to their less pronounced insulin deficiency compared with SAID and SIDD (Table 3). Glycaemic deterioration may thus suggest a milder progression of disease for SIRD, MOD and MARD. However, the higher risk for several complications in SIRD clearly indicates the need for treatment intensification addressing CKD, CVD and NAFLD (e.g. by sodium–glucose cotransporter 2 inhibitors [SGLT2is] and glucagon-like peptide-1 receptor agonists [GLP-1RAs]), as well as targeting insulin resistance (e.g. by future insulin sensitisers) (Fig. 1). Given the proinflammatory profile and the high risk of complications in SIRD, novel therapies targeting inflammatory pathways, as developed for people at high cardiovascular risk [54, 55], could also be considered in the future. Initially, lifestyle modification and metformin are sufficient for treating MOD and MARD. Nevertheless, MOD may specifically benefit from weight loss intervention by hypo-energetic diets and drugs, whereas MARD may be better treated by nutrition avoiding further ageing-related sarcopenia (Fig. 1).

**Table 3:** Novel diabetes subgroups: glucose-lowering therapy in cohort studies and response to therapy in ADOPT

Diabetes subgroup	Therapy in cohort studies	Response to therapy in ADOPT	Comment
SAID	Most frequent use of insulin and lowest use of metformin at baseline [6, 29] Shortest time to sustained insulin use [6]	Not analysed in the context of novel diabetes subgroups	Findings are in line with the established treatment for type 1 diabetes and LADA
SIDD	Most frequent use of metformin at baseline [8, 29] Frequent use of insulin at baseline and short time to sustained insulin use, although less pronounced than for SAID [6] Shortest time to treatment with oral medication other than metformin and longest time to reach HbA_1c_ treatment goal [6]	Initial treatment response best with sulfonylureas but highest HbA_1c_ increase thereafter with sulfonylureas	Data are in line with the low beta cell reserves in this subgroup
SIRD	Most frequently treated with metformin or without glucose-lowering therapy [6] Evidence for higher insulin use later after diabetes diagnosis [29]	HbA_1c_ benefit with thiazolidinedione therapy	Findings are plausible given the pronounced insulin resistance and high prevalence of NAFLD in SIRD
MOD	Most frequently treated with metformin or without glucose-lowering therapy [6] Lowest baseline use of insulin [29]	Initial treatment response best with sulfonylureas but highest HbA_1c_ increase thereafter with sulfonylureas	Data indicate a mild form and mild progression of diabetes
MARD	Most frequently treated with metformin or without glucose-lowering therapy [6] Low cumulative incidence of treatment with oral medication other than metformin or of sustained insulin use [6]	HbA_1c_ benefit with sulfonylurea therapy, limited to about 2 years, vs metformin and thiazolidinedione treatment	Data indicate a mild form and mild progression of diabetes

Data for response to therapy in ADOPT are from a secondary analysis of the trial [28], which randomised newly diagnosed, drug-naive individuals with type 2 diabetes to metformin, sulfonylurea (glibenclamide) or thiazolidinedione (rosiglitazone) monotherapy

LADA, latent autoimmune diabetes of adults

Currently, it is not clear whether our knowledge on mechanisms (and adverse effects) of these drugs will translate into subgroup-specific treatment benefits [56]. However, the large number of ongoing RCTs using novel therapeutic agents targeting insulin secretion, insulin resistance, liver metabolism and other mechanisms that differ between the subgroups holds promise for precision healthcare [57].

So far, only the Look AHEAD (Action for Health in Diabetes) study analysed the differential response to lifestyle intervention [37]. Individuals with type 2 diabetes were allocated to four subgroups, which are not directly comparable with the previously described subgroups [6, 26] due to differences in clustering variables. Randomisation to intensive lifestyle intervention was associated with increased cardiovascular risk in the subgroup characterised by the poorest glucose control and most frequent use of insulin [37]. Thus, subgroups may differ in their response to non-pharmacological treatment, emphasising the differential need for pharmacological treatment intensification to prevent diabetes-related complications.

## Methodological aspects and open questions

The text box above gives an overview of key gaps in our current knowledge, resulting open questions, and future directions in this field; some of the methodological aspects are also briefly discussed here. Any effort made concerning reclassification into subgroups has a strong conceptual appeal because it is easy to communicate and implement once RCTs have shown subgroup-specific differences to non-pharmacological and pharmacological interventions. However, this approach assumes a certain degree of homogeneity within, and clear differences between, subgroups, whereas in reality the characteristics of individuals from different clusters partially overlap [3]. The subgroup approach is also limited by the fact that subgroup assignment requires the availability of all clustering variables. Finally, the utility of subgroups depends on their stability. An analysis in the GDS demonstrated that 23% of the study participants migrated into a different subgroup within the first 5 years after the diagnosis of diabetes [26].

An alternative strategy in precision diabetology may be based on statistical models using continuous risk factors [28, 58, 68]. In a secondary analysis of RCTs [28], age at diabetes diagnosis and renal function at baseline were better predictors of disease progression than the subgroup assignment according to Ahlqvist et al [6]. Thus, specific phenotypic measures to predict glycaemic progression, onset of complications and treatment response could be used to optimise diabetes care in an individualised approach. The risk assessment could be updated regularly to take into account disease progression, with corresponding treatment changes. However, these models would only be useful for optimising one specific outcome such as glycaemic progression or the development of a predefined complication unless they were a priori designed to predict a composite endpoint comprising different outcomes based on the patients’ preferences. Currently, such an approach remains challenging because it requires a huge amount of individual-participant data to develop the underlying models.

One general criticism of the aforementioned reclassification strategies refers to their use of phenotypic data that depend on disease progression, lifestyle and medication and therefore necessitate regular adaptation. In contrast, genotypic data are stable over time and are more likely to be related to causal mechanisms [22]. However, the proportion of diabetes risk that can be explained by environmental risk factors is still greater than the proportion that can be attributed to known genetic risk variants. This means that people with large differences in genetic risk scores show minor phenotypic differences that can be overcome by modifying exogenous risk factors [69, 70]. Currently, it is unknown which of the two approaches or alternatively a combination of both phenotypic and genotypic reclassification would provide the best benefit.

Irrespective of all methodological and practical issues, it is important to emphasise the following points: (1) clinical decision making is always binary at the end (i.e. resulting in the decision to treat or not to treat and in the selection of certain non-pharmacological or pharmacological interventions) and (2) any approach to reclassify diabetes must result in diabetes prevention and care superior to that received under the established classification. The ongoing initiative of the ADA and the EASD on precision medicine in diabetes will provide a detailed roadmap for future studies and application of tailored diagnostics, prevention and treatment on the road to precision diabetology [13].

## Conclusions

The heterogeneity of diabetes, particularly type 2 diabetes, is evident from differences in multiple pathophysiological and clinical features. Recent studies provided novel insights into interindividual differences by clustering people with diabetes into five subgroups, which are reproducible and associated with different trajectories in disease progression and onset of diabetes-related complications including CKD, retinopathy, CVD, NAFLD and neuropathies. Based on the current evidence, it is possible to propose innovative stratified prevention and treatment approaches at least for some of these subgroups (Fig. 1). However, the ultimate test of the utility of precision diabetology will require RCTs to demonstrate whether the probability-based assignment to subgroups and subsequent subgroup-specific prevention or treatment is indeed superior to that proposed by the current guidelines. Furthermore, future studies should address methodological issues, in particular on the best precision diabetology approaches, and also uncertainties regarding the transethnic generalisability of the current findings.

## Supplementary Information

Figure slide (PPTX 230 kb)

ESM (PDF 144 kb)

## Abbreviations

ADOPT: A Diabetes Outcome Progressive Trial
AHEAD: Action for Health in Diabetes
ANDIS: All New Diabetics in Scania
ANGPTL8: Angiopoietin-like protein 8
CAN: Cardiovascular autonomic neuropathy
CASP-8: Caspase-8
CKD: Chronic kidney disease
DSPN: Distal sensorimotor polyneuropathy
EN-RAGE: S100 calcium-binding protein A12
GDS: German Diabetes Study
GLP-1RA: Glucagon-like peptide-1 receptor agonist
hsCRP: High-sensitivity C-reactive protein
MARD: Mild age-related diabetes
MOD: Mild obesity-related diabetes
NAFLD: Non-alcoholic fatty liver disease
PRS: Polygenic risk score
SAID: Severe autoimmune diabetes
SGLT2i: Sodium–glucose cotransporter 2 inhibitor
SIDD: Severe insulin-deficient diabetes
SIRD: Severe insulin-resistant diabetes
VNDS: Verona Newly Diagnosed Type 2 Diabetes Study
