# Supplementary material for: A novel diabetes typology: towards precision diabetology from pathogenesis to treatment
Source: Diabetologia. 2022 Jan 4;65(11):1770–81. doi: 10.1007/s00125-021-05625-x (PMC9522691; doi:10.1007/s00125-021-05625-x)
Supplement: Supplementary file 1 — (PPTX 230 kb) [file 125_2021_5625_MOESM1_ESM.pptx]

## Slide 1
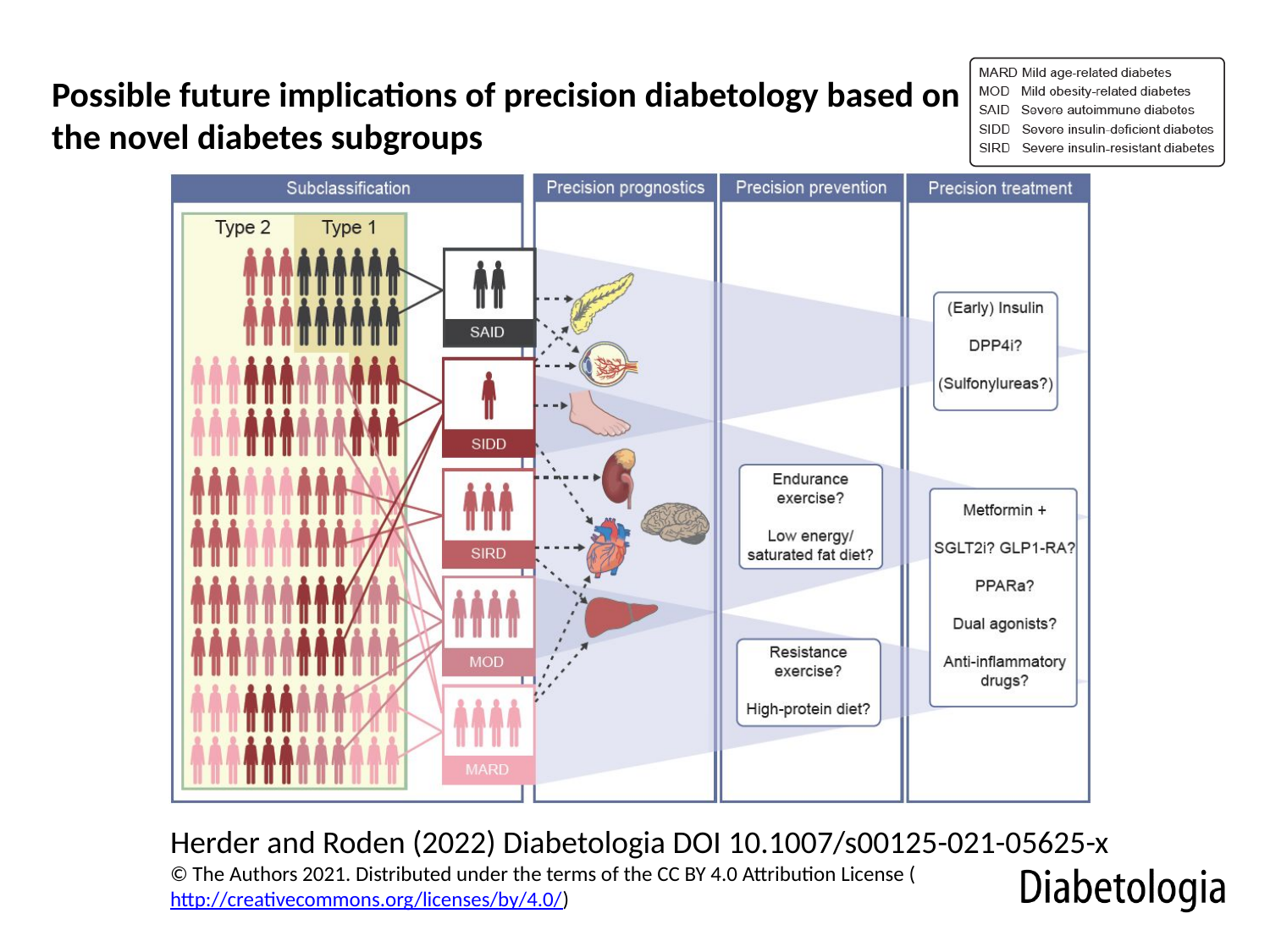

Possible future implications of precision diabetology based on the novel diabetes subgroups
Herder and Roden (2022) Diabetologia DOI 10.1007/s00125-021-05625-x
© The Authors 2021. Distributed under the terms of the CC BY 4.0 Attribution License (http://creativecommons.org/licenses/by/4.0/)
