# Supplementary material for: A novel diabetes typology: towards precision diabetology from pathogenesis to treatment
Source: Diabetologia. 2022 Jan 4;65(11):1770–81. doi: 10.1007/s00125-021-05625-x (PMC9522691; doi:10.1007/s00125-021-05625-x)
Supplement: Supplementary file 2 — (PDF 144 kb) [file 125_2021_5625_MOESM2_ESM.pdf]

ESM Table 1. Evolution of the classification of the main diabetes types.

Author	Observation	Reference
Etienne Lancereaux, 1880	Suggestions that people with diabetes may have different phenotypes preceded the discovery of insulin probably by centuries. Lanceraux clearly formulated and published a concept of separating diabetes types according to fat mass into **“diabète maigre” (lean diabetes)** and **“diabète gras” (fat diabetes)**.	[1]
Wilhelm Falta, 1931	He introduced the concept of two diabetes types, **“insulärer” (insulinar)** and **“insulinresistenter” (insulin resistant) diabetes** along with a complex diet plus insulin tolerance test to separate both diabetes types.	[2]
Harold Himsworth, 1936	He proposed two types of disease that can be distinguished as causing the symptom-complex of diabetes mellitus: the **insulin-sensitive type**, caused by deficiency of insulin, and the **insulin-insensitive type**, apparently due not to lack of insulin, but to lack of an unknown factor, which sensitises the body to insulin. He further described a glucose/insulin test for distinguishing between these diabetes types and suggested that these types might also require different diet treatments.	[3]
Draper and colleagues,1940	These authors applied somatotyping, introduced by W. H. Sheldon, which scores a humans´s physique using a standard photograph against a grid, to find two somatotypes, referred to as **Group I** and **Group II**.	[4]

Robert D. Lawrence, 1946	He provided one of the earliest descriptions of a rare case of **lipodystrophic diabetes**, suggesting that some types may be due primarily to disturbances of fat-metabolism produced by other hormonal influences than insulin. He also separated a **mild obese type**, which never develops ketosis, from an **insulin deficient type**.	[5]
John Lister and colleagues, 1951	These authors combined the Himsworth sensitivity test with Sheldonian somato-typing to describe two broad diabetes groups: the **young thin, non-arteriosclerotic group** with normal blood pressure and usually an acute onset to the disease, and **the older, obese, arteriosclerotic group** with hypertension and usually an insidious onset, which they provisionally designated **type I** and **type II**.	[6]
WHO, 1965	An expert committee came to the conclusion to classify diabetes simply based on age at diagnosis into **4 classes**: **infantile/childhood** (0–14 years), **young** (15–24 years), **adult** (25–64 years) and **elderly** ($\geq$ 65 years), but further also mentioned juvenile-type (requiring insulin and prone to ketosis), brittle, insulin-resistant (reserved for cases requiring >200 units of insulin daily), gestational, pancreatic, endocrine and iatrogenic diabetes forms.	[7]
Andrew G. Cudworth, 1976	He re-introduced **type 1** and **type 2 diabetes** based on Lister´s work and worked on an improved description of autoimmune diabetes.	[8]

US National Diabetes Data Group, 1979	This data group produced a consensus document standardising the nomenclature and definitions for diabetes mellitus, which was endorsed by WHO in 1980 and updated in 1985 and 1994. These classifications included two major classes of diabetes: **insulin dependent diabetes mellitus (IDDM),** or **type 1**; and **non-insulin dependent diabetes mellitus (NIDDM)**, or **type 2**. The 1985 report omitted the terms "type 1" and "type 2", but retained the classes IDDM and NIDDM, and introduced a class of **malnutrition-related diabetes mellitus (MRDM).**	[9-12]
Expert Committee on the Diagnosis and Classification of Diabetes Mellitus, 1997	An international expert committee released a report with new recommendations for the classification and diagnosis of diabetes. The major changes from the 1979 report were: the preferred use of the terms **"type 1"** and **"type 2"** and a simplified diagnostic test based on 2 fasting plasma glucose measurements with a lower cutoff value of 126 mg/dl.	[13]
McCarthy, 2017	He proposed an alternative **"palette" model**, which is centred on a molecular taxonomy focusing on positioning an individual with the major pathophysiological processes that contribute to diabetes risk and progression. Of note, this model anticipates that many individuals might have multiple parallel impairments that affect several of these processes.	[14]

This table focuses on previous attempts for a classification of diabetes, without addressing the history of defining so-called secondary and gestational diabetes forms. Parts of this summary are based on a previous investigation by Edwin A. M. Gale [15].

1. Lancereaux E (1880) Le diabete maigre, ses symptomes, son evolution, son pronostic et son traitement. Union Medicale 29:161

2. Falta W, Boller R (1931) Insularer und insulinresistenter Diabetes. Klin Wochenschr 10:438-443

3. Himsworth HP (1936) Diabetes mellitus: Its differentiation into insulin-sensitive and insulin-insensitive types. Lancet 227(5864):127-136. doi.org/10.1016/S0140-6736(01)36134-2

4. Draper G, Dupurtuis CW, Caughey JL (1940) The differentiation by constitutional methods between pancreatic diabetes and diabetes of pituitary origin. Trans Am Assoc Phys 55:146–53.23

5. Lawrence RD (1946) Lipodystrophy and hepatomegaly with diabetes, lipaemia, and other metabolic disturbances; a case throwing new light on the action of insulin. Lancet 1(6403):724

6. Lister J, Nash J, Ledingham U (1951) Constitution and insulin sensitivity in diabetes mellitus. Br Med J 1951;1(4703):376–379. doi: 10.1136/bmj.1.4703.376

7. WHO Expert Committee on Diabetes Mellitus & World Health Organization (1965). Diabetes mellitus: report of a WHO Expert Committee [meeting held in Geneva from 24 to 30 November 1964]. World Health Organization. https://apps.who.int/iris/handle/10665/38442

8. Cudworth AG. The aetiology of diabetes mellitus. Br J Hosp Med 1976;16:207–16

9. National Diabetes Data Group (1979) Classification and diagnosis of diabetes mellitus and other categories of glucose intolerance. Diabetes 28:1039–1057

10. World Health Organization Expert Committee on Diabetes Mellitus (1980) Second Report. Technical report series 646. WHO, Geneva

11. World Health Organization Study Group on Diabetes Mellitus (1985) Technical report series 727. WHO, Geneva

12. Prevention of diabetes mellitus (1994) Report of WHO Study Group. Technical Report Series 844. WHO, Geneva

13. Report of the Expert Committee on the Diagnosis and Classification of Diabetes Mellitus (1997) Diabetes Care 20:1183-1197. doi.org/10.2337/diacare.20.7.1183

14. McCarthy MI (2017) Painting a new picture of personalised medicine for diabetes. Diabetologia 60(5):793-799. doi: 10.1007/s00125-017-4210-x

15. Gale EAM (2013) Is type 2 diabetes a category error? Lancet 381:1956–57. doi: 10.1016/S0140-6736(12)62207-7
